# The Role of Biosecurity in the Control of *Campylobacter*: A Qualitative Study of the Attitudes and Perceptions of UK Broiler Farm Workers

**DOI:** 10.3389/fvets.2021.751699

**Published:** 2021-12-21

**Authors:** Alexandra Royden, Robert Christley, Alison Prendiville, Nicola J. Williams

**Affiliations:** ^1^Department of Livestock and One Health, Institute of Infection, Veterinary and Ecological Sciences, University of Liverpool, Cheshire, United Kingdom; ^2^London College of Communication, University of the Arts London, London, United Kingdom

**Keywords:** *Campylobacter*, biosecurity, food safety, broiler chicken, interview, compliance, qualitative

## Abstract

*Campylobacter* is the leading cause of human bacterial diarrhoeal disease worldwide, with poultry meat products contributing to a large proportion of cases. Due to the ubiquitous presence of *Campylobacter* in the poultry farm environment, biosecurity is the main area for intervention to prevent colonisation of commercial broiler chicken flocks. However, research has repeatedly demonstrated that farmers' uptake of biosecurity recommendations is often poor. This study explored farmers' attitudes towards biosecurity and identified barriers to effective implementation of biosecurity protocols. Semi-structured interviews were conducted with 1–3 members of staff on each of 16 broiler farms; 6 owned by, and 10 contracted to, 3 different UK poultry integrators. In total, 28 interviewees participated, including farm owners, managers, and workers, with a range of industry experience. Thematic analysis of the interviews revealed high levels of recognition amongst broiler farmers of the importance of *Campylobacter* and the responsibility of the whole farm-to-fork chain within the poultry industry to reduce *Campylobacter* contamination of chicken meat for the benefit of public health. Participants' self-reported awareness and implementation of biosecurity has improved significantly following the industry-wide focus on *Campylobacter* control. However, there are frustrations with the industry's approach to tackling *Campylobacter* and the heavy burden of responsibility that has been put on interventions at the farm-level. There was also scepticism amongst participants as to the effectiveness of current biosecurity measures in the reduction of *Campylobacter*. Nevertheless, the interviewees' recognition of the benefit of improved biosecurity on broiler health and welfare and other important targets, such as reducing antimicrobial usage, leaves a legacy of which the UK broiler industry can be proud. There is scope for further farmer education about the evidence supporting biosecurity interventions, particularly in the control of *Campylobacter*, and a need to establish more effective channels of communication. Furthermore, to give all players within the industry agency and investment in industry targets, contributions from all levels should be permitted in the design of future biosecurity interventions. Biosecurity compliance may be improved through collaborative efforts, such as participatory and co-design practises, to facilitate knowledge co-creation and exchange.

## Introduction

*Campylobacter* is the leading cause of human bacterial diarrhoeal disease worldwide. Poultry meat and products are estimated to account for ~70% of human campylobacteriosis cases in the UK, due to the consumption of undercooked meat or cross-contamination of raw meat within the kitchen (1). Due to the ubiquitous presence of *Campylobacter* spp. in the poultry farm environment, biosecurity practises have been highlighted as the main area for intervention to prevent the spread of *Campylobacter* into and between broiler houses and the subsequent colonisation of commercial broiler chicken flocks (2–5). “Biosecurity” encompasses all hygiene practises that are put in place to ensure the risk of a disease occurring or spreading is minimised (6). Biosecurity measures are designed to prevent the introduction and spread of disease-causing organisms into a flock or herd (7, 8) and have been shown to be key in the prevention of disease in poultry units (9, 10). Controlling *Campylobacter* at the farm-level is crucial to reduce the level of *Campylobacter* entering processing plants and the public health risk to consumers (11). The consistent application of biosecurity measures is essential for the success of all types of animal production, including to prevent disease introduction and reduce production and financial losses that may occur following infection of a herd or flock (7, 8). However, research has repeatedly demonstrated that compliance with biosecurity protocols is poor, despite serious and potentially economically devastating consequences (8). Moreover, there has been little research regarding attitudes and perceptions of biosecurity measures with people working on broiler farms, particularly within the UK poultry industry.

In 2010, the Food Standards Agency (FSA) and the UK poultry industry set a joint target to reduce *Campylobacter* in chicken meat at retail; aiming to reduce the prevalence of the most contaminated chickens (>1,000 cfu/g) to below 10% at the end of the slaughter process, initially by the end of 2015 (12). From 2014 to 2018, the FSA conducted UK-wide surveys of *Campylobacter* contamination on fresh chickens at retail. Over this period, overall prevalence reduced from 73.2 to 40.9%, and the percentage of chickens contaminated with >1,000 cfu/g reduced from 19.7 to 3.8% (13, 14). This focus on the reduction of *Campylobacter* followed the introduction of the UK National Control Programme (NCP) for *Salmonella* in meat chickens. This resulted in the introduction and enforcement of biosecurity measures on broiler farms to ensure that the percentage of meat chicken flocks remaining positive for *Salmonella enterica* serotype Enteritidis and *S*. Typhimurium was <1% by the end of 2011, as set out in Regulation (EC) No 646/2007 (15). The Red Tractor Assurance Scheme (16), which represents 1,097 UK broiler chicken assured members and 90% of UK broiler production (17), amended their standards in 2011 to improve biosecurity requirements amongst their members (18). This included the implementation of a designated hygiene area, either demarcated with a physical barrier or a clearly marked area, and footwear change and hand sanitisation at shed entry (18). In 2014, Red Tractor further increased focus on farm biosecurity in response to concerns over *Campylobacter* (17, 18). Requirements introduced included defined biosecure areas for farm and shed entry and equipment cleansing, disinfection of vehicle wheels and equipment at farm entry, footdips, physical barriers, and footwear changes at entry to each biosecure area, inclusion of biosecurity requirements during flock depopulation events and the requirement of all staff to hold a “Poultry Passport,” which includes a biosecurity training module. Further auditing and penalty measures were also introduced to ensure compliance (18). In the same year, the FSA first “named and shamed” supermarkets over the levels of *Campylobacter* in their chicken meat (19), increasing the visibility of the high levels of *Campylobacter* contamination of UK-produced chicken meat and the progress of industry reduction targets. One outcome of these targets has been a rapid evolution in on farm biosecurity measures enforced by poultry assurance schemes and integrators, changing the working practises of farm workers throughout the UK broiler industry in a short period of time.

This research aims to explore how broiler farm workers are responding to these recent industry targets and culture changes. The success of new control initiatives depends upon compliance on-farm, and it is crucial to understand the motivations of people working on broiler farms to enable the development of achievable strategies and suitable biosecurity measures appropriate for the UK poultry industry. Previous studies of both agricultural and domestic animal sectors have found co-design and participatory approaches to be fundamental in encouraging biosecurity compliance (20–22) and the relevance of these strategies to the UK poultry industry will be explored in more depth throughout this study.

This study aims to elicit farmers' attitudes and perceptions to biosecurity, identify barriers for maintaining biosecurity protocols, and to investigate risky behaviours associated with biosecurity breaches and the introduction of *Campylobacter* into poultry houses. These topics will be explored both in relation to general biosecurity measures and in the context of controlling *Campylobacter* on broiler chicken farms.

## Materials and Methods

### Selection of Interview Participants

Farms were recruited from one major poultry integrator (Integrator 1), who nominated six company-owned and eight independent-contracted farms, representing a range of internal biosecurity audit scores and *Campylobacter* testing results. A further two independent farms contracted to two other poultry integrators (Integrators 2 and 3) were recruited by word-of-mouth. All participants were approached directly by telephone or email to request participation and arrange a suitable time and location for the interview. All farms were commercial, intensive, indoor broiler chicken farms in mainland UK. Farms rearing slow-growing broiler chicken breeds and/or with free-range farming practises were excluded from the study. These farms were excluded as they represent a small minority of UK broiler chicken production. There is also a significant difference in biosecurity practises between housed and free-range broiler flocks meaning it would not be possible to explore similar experiences with biosecurity practises with staff on these differing sites.

### Ethics

The study protocol was reviewed and approved by The University of Liverpool Veterinary Research Ethics Committee (Reference VREC478). All participants were informed that participation would be anonymous with all data anonymised prior to publication, so that farms and farmers could not be identified in any published results. Permission to record the interview was sought at project outset with the integrator and prior to the interview with the participant as it was considered a vital component of the qualitative interview process to facilitate subsequent data analysis.

### Interview Design

The interviews were undertaken by a single interviewer. Interviews were semi-structured and used a topic guide (Table 1) to ensure key areas were covered in each interview. However, the interview was participant-led, with the order of the interview determined by the participant(s) and additional relevant topics pursued as they arose. Questions were non-leading and phrased to encourage participants to communicate their personal views and anecdotes. The interview guide was reviewed and revised following an initial pilot interview.

**Table 1:** Interview topic guide.

**Topic headings**	**Areas explored**
Profile of individual	- Education, length of service and motivation
Training and feedback on biosecurity	- Training (and type of) in biosecurity - Feedback on biosecurity
Perceptions and Implementation of Biosecurity	- Definition and importance of biosecurity - Current biosecurity measures - Perceptions of biosecurity standards- Difficulties in practising biosecurity - Improvements to make biosecurity easier- Incentivisation to follow biosecurity
Responsibility for biosecurity and control of *Campylobacter*	- Responsibility for biosecurity and *Campylobacter-*status of flocks - Trusted sources of information
Future options and challenges	- Opinions on the future of biosecurity and the control of *Campylobacter* in the UK broiler chicken industry

### Thematic Data Analysis

All interviews were transcribed verbatim by a commercial transcribing firm, except the pilot interview which was transcribed by the interviewer. Transcripts were then checked for accuracy and anonymised by the interviewer. Thematic analysis (23) was used to assess the transcripts to highlight minor and major themes. Analysis was inductive with themes developed from the data collected. Initial line-by-line coding of transcripts revealed recurring opinions and subject areas which were assigned as minor themes. Minor themes were linked together and common subject areas exposed and categorised as major themes. Interviews were continued until “sampling-to-saturation,” where there were no new ideas emerging from the interviews and there was little or no change to the codebook (Supplementary Material). All analysis was undertaken using the qualitative data management tool NVivo 12.1.0. Further analysis was carried out looking at how commonly themes occurred and examining minority and majority opinion.

## Results

### Study Population

Sixteen interviews were conducted with between one to three members of staff on 16 broiler farms, six owned by and 10 contracted to three different UK poultry integrators (Integrator 1, 2, or 3). A total of 28 participants, three females and 25 males, took part, including farm owners, managers, and workers, with a range of experience in the broiler industry. Further details of the interviewees and farms involved in the interviews are included in Table 2. The length of the interviews ranged in time from 33 min to 1 h 44 min (Mean = 48 min; Median = 44 min). A pilot interview took place in May 2016, with the rest conducted between June 2017 and January 2018. The transcript from the pilot interview was reviewed in detail and considered to be of acceptable quality to be included in the overall analysis.

**Table 2:** Details of 28 interviewees and 16 farms participating in 16 interviews in May 2016 and between June 2017 and January 2018.

**Farm no**.	**Integrator 1, 2 or 3 [Independent Farm (I)/Company-Owned Farm (C)]**	**No. of houses on site (approximate total no. of broilers per flock cycle)**	**No. of participants in interview [male (M)/female (F)]**	**Role [Owner (O), manager (M), assistant manager (AM), general worker (W), spouse (S)]**
1	1 (C)	12 (310,000)	2 (M)	M and AM
2	1 (I)	11 (400,000)	3 (M)	M, AM, and W
3	1 (I)	4 (170,000)	1 (M)	O (also M)
4	1 (I)	6 (210,000)	2 (M+F)	M and AM
5	1(C)	9 (290,000)	2 (M)	M and AM
6	1 (I)	4 (130,000)	2 (M)	O and M
7	1 (I)	4 (210,000)	2 (M)	O and M
8	1 (I)	4 (130,000)	2 (M)	O and W
9	1 (C)	8 (210,000)	2 (M)	M and AM
10	1 (I)	8 (320,000)	1 (M)	M
11	1 (C)	8 (300,000)	2 (M)	M and AM
12	1 (C)	8 (290,000)	1 (M)	M
13	1 (C)	8 (280,000)	2 (M)	M and AM
14	1 (I)	4 (200,000)	1 (F)	M
15	2 (I)	4 (130,000)	2 (M+F)	O (also M) and W (also S)
16	3 (I)	3 (80,000)	1 (M)	O (also M)

### Themes Identified

Through thematic analysis of the transcripts, minor themes were found to link to overarching major themes. Six major themes and seven minor themes were identified and will be discussed in more detail below:

1. *Campylobacter* in Vogue
2. The Importance of Biosecurity
  a. The Legacy of *Campylobacter* Control
3. Scepticism and Controversy
  a. About *Campylobacter*
  b. About *Campylobacter* Control
4. Biosecurity Compliance
  a. Requirement and Enforcement
  b. Other Contributing Factors
Biosecurity Issues and Improvements
  a. Specific Biosecurity Issues
  i. Control Room Barriers
  ii. External Site Visitors
  iii. Partial Flock Depopulation (“Thinning”)
  b. Potential Improvements
6. Power and Responsibility

### *Campylobacter* in Vogue and the Legacy of *Campylobacter* Control

Since the industry introduced targets to reduce *Campylobacter* in broiler chickens, participants believed that there had been an improvement in on-farm biosecurity practises and farmers' understanding of their importance:

“*Obviously, as farms, by doing things like the […] barriers and other biosecurity measures, we are improving the health of the chickens, generally, anyway. So, therefore, how can you knock the biosecurity? Obviously, it's had a benefit on the health of the chickens so far. Even if you totally forget the Campylobacter side of it, there are definite improvements that we have done so that hasn't been a bad exercise.” – Independent-Contracted Farm Owner and Manager*

Interviewees described “*a definite knock-on effect,”* whereby the increase in biosecurity and the drive to reduce *Campylobacter* had improved other aspects of broiler chicken production, including overall flock health and performance. There was repeated emphasis that biosecurity was important for the overall health and performance of the flock:

“*Listen. Biosecurity is just as important as performance. If you haven't got biosecurity, you haven't got performance. They go hand-in-hand, and they really do.” – Company-Owned Farm Manager*

There was a prevalent opinion that the focus on *Campylobacter* was “in Vogue” and a fashion and would pass in time:

“*It's very much the thing at the moment. Without a doubt, in a period of time, it won't be highlighted. There might be something else that comes on the- You know, there might be another thing that is highlighted, and we'll have to concentrate on that. Salmonella, that's a strange one…A few years ago, that was the thing they were concentrating on.” – Independent-Contracted Farm Owner and Manager*

The focus on the reduction of *Campylobacter* was said by interviewees to be a recent development. Interviewees commented that they had not heard of *Campylobacter* until recently and called it a “*new problem.”* Others believed that, until recently, either “*there wasn't an issue with Campy”* or felt that it had been “*kept quite quiet.”* The tightening of biosecurity measures was described as “*a big sea-change”* within the broiler chicken industry and a current prominent focus of the integrators. Interviewees cited the public “naming and shaming” in the media of the prevalence of *Campylobacter* in individual supermarkets' retail chicken as the event that kick-started industry attention towards *Campylobacter*.

The time frame for the introduction of increased biosecurity measures on farms was estimated by many to have been within the last 2 to 5 years, with many citing the lack of specific biosecurity measures prior to this as evidence of how quickly the focus on biosecurity to reduce *Campylobacter* has spread through the industry. On-farm biosecurity was framed as “before-*Campylobacter*” and “after-*Campylobacter*.” Interviewees mentioned that “before,” farms only had foot dips at the entrance to each shed, which were “*virtually optional,”* and there was no requirement for extensive personal-protective equipment (PPE) or shed-specific clothing and equipment. Whereas “after,” strict requirements to follow enforced biosecurity protocols were introduced. An improvement in biosecurity around “thinning and catching” (“partial and final flock depopulation”—discussed in more detail below) was also cited to have been introduced in this period.

Participants highlighted that *Campylobacter* control was a multifactorial problem, with many factors that need to be controlled to minimise risk and reduce levels of colonisation. It was commented that this is why *Campylobacter* is a more frustrating pathogen to control than *Salmonella*:

“*[Campylobacter]'s not going to be like Salmonella where there's a silver bullet, you can just- you can solve it.” – Independent-Contracted Farm Manager*

Comparisons were drawn with recent efforts in the poultry industry to tackle *Salmonella* and how the focus on *Salmonella* was replaced by *Campylobacter*. Interviewees believed that a solution would be found to reduce Campylobacter to acceptable levels, with some adding that there would then be another problem to tackle as “it does seem to be a never-ending battle with something”.

There was a feeling amongst participants that even if *Campylobacter* could not be eradicated from broiler farms, this focus within the industry had left a legacy and had a positive impact on broiler production. Furthermore, improved biosecurity was argued to be facilitating the poultry industry's ongoing targets to reduce antibiotic usage, which was understood to be a positive change. All participants understood how important biosecurity was in the prevention and control of all infectious pathogens, including *Campylobacter*. Participants felt that the presence of *Campylobacter* in a flock was an indicator of poor biosecurity and believed that farms with poor biosecurity were more likely to have *Campylobacter*-positive flocks.

### Power and Responsibility

Participants believed that the whole poultry industry, from farm to fork, had a responsibility for the reduction of *Campylobacter*, with some framing this as a moral obligation:

“*I think as an industry we have an ethical responsibility to provide a food safe product to the consumers.” – Independent-Contracted Farm Manager*

None of the interviewees suggested that farms did not have a role to play in reducing *Campylobacter* in chicken meat and, as previously explained, believed that the recent improvement in biosecurity was beneficial to the industry as a whole. However, interviewees did not believe that *Campylobacter* negatively affects broilers but is only of concern to human health. A small number of participants expressed the opinion that if *Campylobacter* did have a detrimental effect on chicken health and welfare then it would have been eradicated from broiler farms:

“*I think I probably shouldn't say this, but when somebody says, “If Campylobacter affected chickens, it would have been sorted out years ago.” It's true, it would have been, but the fact that it has no detrimental effects to the chicken, is why…I was going to say, we don't have to worry about it, but our job is to grow the chickens as well as we can, to the best standard and welfare as possible.” – Independent-Contracted Farm Owner*

There was a common view that farms have been unfairly targeted and more could be done in other areas of production. Many participants did not believe that *Campylobacter* could be eradicated from broiler chicken flocks. It was felt that improvements could be made during processing to reduce the levels of *Campylobacter* on broiler carcasses and that by introducing more interventions in slaughterhouses, this would reduce the burden of responsibility on farms and may even eliminate *Campylobacter* from retail chicken.

There were several controversies and frustrations expressed by participants surrounding the biology of *Campylobacter* and their understanding of its transmission. Each farm had specific issues which they believed were the cause of *Campylobacter* on their farm, for example the ventilation, the weather or climatic factors, pests and public/vehicular access routes. Often blame was passed onto others, such as the breeder flocks, hatcheries, feed mills or catchers. Whether or not it was scientifically plausible, blame was largely shifted onto something that was out of the individual's control.

Whilst many of the interviewees thought increased biosecurity was important, some were sceptical as to its effectiveness in the reduction of *Campylobacter* and others did not believe that biosecurity was the solution to controlling *Campylobacter*. Participants explained how they consistently applied the correct biosecurity measures and flocks would test positive or the results of testing differed between flocks. Participants discussed their frustrations with trying to predict when flocks would be *Campylobacter* positive and that this “*appears to follow no patterns.”* Many felt that flocks that “should be” negative would test positive, and those that had suffered biosecurity breaches, either necessary or accidental, would test negative. This participant explained this phenomenon:

“*When you've got good biosecurity, it's got to lower the risk of getting Campylobacter. Then you go to some places, their biosecurity is top notch, new sheds, all very clean everywhere and they've still got very bad Campylobacter.” – Company-Owned Farm Manager*

Some participants believed that the farmers were often ignored in the decision-making process that would ultimately affect their daily lives, as explained by this participant:

“*The conversation we've had now is ten times longer than any- is the only conversation where anybody has asked me any questions about what we do…And, just a point of view. They don't want to know about the little guys. The little guys, the farmers are the guys that actually keep them going. So, for instance, we went to a meeting not long ago and had 50 farmers in the room. Average experience – 10 years each. I'm a bit of an ‘old in the tooth' one now. So that's 500 years of growing chickens experience in that room and not once in a four-hour meeting did the integrator representatives actually ask for anyone's opinion. They told us, and these guys are people who have never actually grown a chicken in their life. So rather than saying, “We've got this problem, this is what we think, has anybody got any ideas?”, [they said,] “This is the problem, this is what we're going to do about it.” They were wrong on so many levels.” – Independent-Contracted Farm Owner and Manager*

Participants expressed a desire for feedback and communication from the integrator about current events and developments in the production chain, including welfare and biosecurity requirements. The integrators were reported to hold considerable power over their farms, particularly financial influence with regards to contracted farms, where they were described as “*the paymaster.”* Interviewees described the increasing pressure put on them by integrators to comply with biosecurity regulations. One contracted farm explained they had considered stopping broiler farming due to the requirement to comply with increasing regulations. Other interviewees from contracted farms commented that they had switched contracts due to the “*dictatorial”* nature of integrators and the high level of supervision and oversight.

### Biosecurity Issues, Compliance, and Improvements

Interviewees were asked to describe the biosecurity measures employed on site and to discuss any specific compliance issues that had arisen in the implementation of these and also suggestions for improvement. All participating farms were part of the Red Tractor assurance scheme which requires adherence to a minimum biosecurity standard. The common biosecurity measures employed across all farms interviewed included, but were not limited to, restricted and monitored access of the farm perimeter, an anteroom at the entrance to the broiler house containing a physical barrier delineating a biosecure area, widespread use of disinfectant footbaths, farm and broiler house-specific clothing and footwear, and rigorous policies preventing the introduction and spread of disease. Of the common biosecurity measures implemented on-farm, issues with specific biosecurity measures and flock events repeatedly arose during interviews: the control room barriers, external site visitors, and partial flock depopulation.

### Control Room Barriers

Commercial poultry houses frequently separate the flock from the outside world with an “anteroom” or “control room,” a room within the house that must be entered by staff and visitors before entering the main area housing the flock. Anterooms are frequently split into two areas by a barrier; a defined demarcation zone to change boots, with the area closest to the door giving access to the birds being considered “clean.” Under current Red Tractor standards, the type of separation between the contaminated and the clean areas must be a permanent or removable (for cleaning purposes) physical barrier, such as low wall (16, 18, 24, 25).

The usefulness of such barriers was questioned by participants. There was a great deal of frustration amongst participants that the type of barrier within the control room had changed multiple times in a short period of time, which fueled a common belief that the industry did not know how to control *Campylobacter* but needed to be seen to be doing something. Participants sometimes used lay understandings, often at odds with current scientific “facts,” to explain their attitudes to, and behaviour regarding, recommended biosecurity practises. For example, not observing control room barriers was due to a perception that barriers fail to prevent *Campylobacter* colonisation and a lack of evidence to the contrary. Interviewees also commented on the practicality and usefulness of some of the required biosecurity measures and were scornful of the people, “*sat in an office somewhere,”* who introduced them. In addition, the cost of having to change the barriers multiple times to comply with regulations was a source of frustration for the independent farms. The barrier was described as a health and safety risk and expected to eventually be removed from sheds for this reason.

### External Site Visitors

Many participants felt that external site visitors, such as relief staff and external maintenance staff, did not comply with biosecurity protocols or use site-specific clothing and equipment and had to be “*babysitted.”* Participants felt that visitors did not understand the importance of biosecurity and that because “*they haven't got the ownership thing,”* they did not feel that it was important to follow the protocols in place. Participants felt that larger sites with more staff were more difficult to keep *Campylobacter*-free. Vehicular access was a major issue for many participants; compliance with and the effectiveness of wheel washing was questioned, and participants believed that drivers were a biosecurity risk.

### Partial Flock Depopulation (Thinning)

During intensive broiler chicken production, a process called “partial depopulation,” also known as “thinning,” takes place. At the beginning of each flock cycle, sheds are stocked with extra birds, some of which are then removed during thinning. This ensures the correct stocking densities are maintained whilst the remaining chickens grow to the desired final slaughter weight before the flock goes for final processing. Thinning is common practise throughout the UK poultry industry; allowing farmers to maximise productivity by utilising available space, whilst ensuring that the birds are kept at the correct stocking density to meet necessary welfare requirements. Poultry “catchers” are employed to collect (“catch”) chickens from farms during flock depopulation events. Catchers are either contracted by farms and poultry companies or employed by an integrated poultry company. Catchers work in groups of 4–6, catching 5,000–6,000 birds per hour during 15 h days of very physical work in tough conditions (26, 27).

Participants mentioned that flocks were stocked at lower densities and thinned less (only once as opposed to two or three times) than they used to be, which was seen to have been introduced primarily as another anti-*Campylobacter* measure. A ban on thinning to help control *Campylobacter* was felt to be a very political issue within the broiler industry, with some integrators keener to implement a ban than others.

“*It doesn't matter what you do, you've got to take the forklift in the shed, you've got to take the modules in the shed and the catcher's got to go in the shed. So, the way around it is simple. Don't thin. The answer to not thinning though, is expensive, because it puts 10p a bird on price on the shelf. And it means we need 20% more growing space in the UK.” – Independent-Contracted Farm Owner and Manager*

There was very little appetite to stop thinning amongst interviewees; the economic impact this would have on broiler production and the need for decreased stocking densities was thought to be too financially devastating. Integrators with lower stocking densities were said to be more in favour of a ban because it would put their competitors at a commercial disadvantage.

On farms testing for *Campylobacter* before and after thinning, many participants commented that flocks often tested positive after thinning. There were very mixed views on the catchers themselves.

“*There are some catchers that don't really care about their own personal hygiene, let alone my biosecurity.” – Company-Owned Farm Manager*

Participants expressed frustration that they follow biosecurity protocols diligently, only to have the catchers enter the sheds during thinning whilst not observing biosecurity restrictions. Some interviewees felt that because catchers are not invested in the farms, they are not invested in upholding the required standards of biosecurity. However, it was understood that catching is highly pressurised and time sensitive and very little can be done to change this process:

“*They try their best, they wash their wellies, they do everything that's feasibly possible. I can't think of anything else that they could do.” – Independent-Contracted Farm Owner*

It was recognised by many that catchers supplied by a company were better at following biosecurity than those on contracts. This was often felt to be because contracted catchers were paid by the bird. Company catchers had more oversight and were easier to hold to account. Some interviewees did not believe that there was any difference between company and contract catchers but just between catching teams. The relationships between the catching team-leader, the catching team and the farm staff were recognised as important parts of whether the team adhered to biosecurity measures and best catching practise.

Participants discussed the impact of stress on broiler health, including gut health. One interviewee described the stressors that can affect a flock during thinning:

“*They're not used to it, they're plodding along having a laugh, and then one day the lights get turned off, they've been taken off feed. Then you've got this big forklift coming in making a racket, all the catchers, put the mods in the sheds, they're not used to that.” – Company-Owned Farm Manager*

Participants believed that increased stress increased susceptibility to *Campylobacter* infection. Although participants discussed other sources of stress that occurred throughout the flock cycle, such as weather and feed changes, the most stressful period was described as thinning. Sources of stress during thinning included feed withdrawal prior to and during thinning and changes in lighting and high and/or unusual noise levels during thinning. Heat was also regarded as a major source of stress, either from the weather or generated by thinning. Some participants felt that if the birds became stressed there was very little that could be done to prevent *Campylobacter* infection and that they were able to predict *Campylobacter*-positive flocks from the occurrence of certain stressors during the flock cycle.

### Biosecurity Compliance and Improvements

A considerable proportion of the interviews was spent discussing the main motivators, which encourage farmers to follow biosecurity, and barriers, which discourage them from implementing the required standards. Broadly these fell into two categories: (i) biosecurity compliance due to requirements and enforcement and (ii) biosecurity compliance, or non-compliance, due to other factors such as time pressures, financial (dis)incentives and personality traits.

There was a high level of acceptance amongst interviewees of the requirement to carry out biosecurity measures. Whether or not the interviewee understood why they were being asked to carry out the biosecurity practise or believed in the effectiveness of the measure, many carried it out simply because it was required:

“*What we're doing at the moment, it doesn't motivate me to be stricter, because what we're doing is what we've been told to do anyway. There's no more that we can do, it's like, if they tell us, “We want you to do it this way.” We'll do it this way, we'll just do what they tell us to do.” – Company-Owned Farm Manager*

Many participants commented that the biosecurity requirements had become a habit and that over time they had got into a routine of practising certain measures, despite the extra time it took to complete some tasks compared to in the past. The additional time-cost to follow biosecurity protocols was mentioned by participants to have added pressure to broiler farming:

“*There's time. When you think we go in the shed and we've got to change wellies, put these overalls on, gloves on, so you're there five, ten minutes in the shed, times that by [no. of sheds]. It just takes a ridiculous amount of time. We've seen like, it takes us a hell of lot longer to walk the birds now, just from all of this coming in. Whatever you've got to do I suppose.” – Independent-Contracted Farm Assistant Manager*

Others admitted that they are less compliant with biosecurity when they enter the shed for a non-routine matter, particularly when addressing issues that might affect welfare:

“*When you've got a breakdown, biosecurity goes out the window. It's the birds' welfare at the end of the day.” – Independent-Contracted Farm Manager*

Participants also confessed that they were worse at adhering to biosecurity protocols if they enter a broiler shed at night in the event of an alarm. Shed-specific PPE and overalls were commonly cited as measures that participants were likely to ignore, notably at night or if the sheds were very hot, for example during chick placement and the first few days of the flock cycle.

Company farms were said by some to be better at biosecurity compliance than independent farms because there was greater oversight and enforcement by integrators.

“*Yeah, the independent sector is probably the worst, because we can just do our own thing.” – Independent-Contracted Farm Owner and Manager*

Interviewees commented that the length of time a person had spent in the industry influenced biosecurity compliance, with those new to the industry more likely to comply than those who had witnessed the evolution of biosecurity within the industry. Personality was also said to influence adherence to biosecurity protocols. Some participants told anecdotes in which one individual was credited with a farm's good or bad record with *Campylobacter*. This was used as an example of how personality and individual differences in behaviour were crucial in *Campylobacter* control. Participants felt that certain stressors, such as staff shortages and lack of time-off, may demotivate some farmers and result in poor biosecurity compliance.

Participants recognised the labour required in policing compliance and the difficulty in ensuring that everyone was consistently adhering to the required measures. Interviewees admitted that they ignored certain biosecurity protocols unless they were being visited or audited. Audits were viewed by interviewees as a way to satisfy management and minimise the level of oversight from their managers. Other participants expressed the view that complying with biosecurity practises was a “*tick box exercise”* to fulfil for auditing purposes. Some felt that there was too much auditing within the industry and described the biosecurity audits as “*a hassle,”* which acted as a drain on time and resources and an unwelcome distraction from necessary farm work.

Participants commented that financial incentives or penalties related to *Campylobacter* testing results may result in more effective and quicker uptake of desired biosecurity measures.

“*It depends on what they're trying to achieve with the biosecurity I suppose. If they're just being told to improve their standards because of Campylobacter, there's no financial penalty at all or risk to their business, they might not see the bigger picture of, “That actually also protects you against all the other diseases as well”.” – Independent-Contracted Farm Manager*

Financial considerations were cited as both a pro and a con with regards to biosecurity compliance. One participant remarked that “*with farmers, most things are financial.”* Independent farms were said to be more resistant to change “*if there's a price tag attached to it.”* The lack of financial incentive to reduce *Campylobacter* and the fact that *Campylobacter* is not seen to detrimentally affect the chickens were cited as reasons for poor biosecurity compliance. This was contrasted to the effort to eliminate *Salmonella* from flocks, where there are financial implications for *Salmonella-*positive flocks, which was said to better motivate farmers to produce negative flocks. However, independent farms were also said to be more likely to comply with biosecurity for the control of *Campylobacter* because they are financially and emotionally invested in their farm and the benefits of compliance include better flock performance and therefore profit. Those who felt that financial incentives would improve biosecurity compliance considered that testing would have to be done by an impartial external party and that considerable manpower would be required to do this.

Many of the interviewed farms undertook routine *Campylobacter* testing. The results of this were seen as a reliable indicator of on-site biosecurity compliance. In addition, some interviewees were part of a *Campylobacter* league table where theirs and other farms' *Campylobacter* results were published every crop. Participants' opinions on public league tables were mixed and largely dependent upon whether farms scored highly or not. Participants who were scoring poorly admitted that this was why they did not like the system, but that if they performed well, they were happy to have their results shared. Some participants were embarrassed by the results of their flocks' *Campylobacter* testing and exhibited a sense of pride that they did not want to be seen near the bottom of the league table. Participants did not like it when their farms slipped down the league table, but this encouraged them to better future results. Those participants who found the league table motivating felt that it improved their job satisfaction and encouraged healthy competition between farms. Others felt it improved collaboration and knowledge exchange between farms:

“*It makes you see where you are from other people, it makes you think what other people are doing and that's where the chatting starts. You talk to other people and find out what they're doing differently." – Independent-Contracted Farm Manager*

Participants explained that a public results table encouraged some people to cheat the system by not sampling correctly or trying different methods to ensure that the submitted swabs would be negative. Participants did not feel that there was a benefit to scoring well but felt that scoring poorly resulted in forms of punishment with the results framed as “*who has been a good boy and who has been a bad boy.”* Participants scoring poorly felt demotivated by their results, especially where they felt they were doing everything that had been asked of them. Some at the lower end of the league table wanted to improve but felt that they were fighting a losing battle. Participants reiterated that *Campylobacter* was very difficult to control and that they were being punished for something that was not their fault.

Interviewees described meetings and training events they had attended on *Campylobacter* and biosecurity measures. These were cited as an important method to improve compliance by helping farmers understand why certain measures had to be implemented.

“*Some people say it's down to whether you're lazy or not, but I think it's down to whether you personally think it makes a difference.” – Independent-Contracted Farm Manager*

Participants felt that training would be beneficial to ensure that people did not only see biosecurity as a measure to reduce *Campylobacter* but as a method to improve the welfare, health and performance of the birds, to reduce the economic impact of an infection and, for contract growers, to protect their business, as explained by this participant:

“*Training? If people actually realised that they're actually helping themselves. Yes, it is a hassle…you shouldn't be looking at it as a Campy benefit, but it might help you reduce the risk of your birds getting infected with something else which will impact on you.” – Independent-Contracted Farm Owner*

There was a general belief that the easier and more practical something was to implement in a broiler farm setting then the more likely people were to follow it.

“*I think the key to improving standards on farms is to make sure it's workable and easy for the people that have to use it every day. There was a discussion around showering in and out of every shed and it just wouldn't be done. I mean, if you got an alarm call at three o'clock in the morning, there's no way the farmer's going to go for a shower and go in and sort it out. It has to be workable.” – Independent-Contracted Farm Manager*

Participants felt that there were very few measures left that could be introduced on farms. Showering was commented upon as the only measure left to be introduced on farms. However, this was not a popular idea, with many believing it to be impractical. It was suggested that as older sheds were replaced, biosecurity would improve across the industry and that new builds should be encouraged or required to comply with gold standard biosecurity practises to improve compliance and achieve industry uniformity. However, there was a prevalent sense of fatalism and defeat that *Campylobacter* was largely out of farmers' control. There was a common view that as *Campylobacter* is a ubiquitous bacterium present in the farm environment it is very difficult to tackle.

## Discussion

This study used semi-structured interviews to elicit the attitudes and perceptions to biosecurity of people working on broiler farms in the context of *Campylobacter*-control. Whilst studies have demonstrated that there is poor correlation between self-reported and observed compliance (24, 28), the main aim of this study was not to quantify biosecurity compliance but to investigate the incentives and barriers to compliance with biosecurity measures. A qualitative approach was thus appropriate for this broad exploratory context and has provided a method for understanding farmers' beliefs regarding the relative importance of biosecurity in different situations, the contexts in which their behaviour might differ, and their perceptions of their role in the control of *Campylobacter*. This qualitative approach revealed that the main barriers to biosecurity compliance included a lack of training and education on biosecurity and scepticism that *Campylobacter* control could be achieved through current biosecurity measures. There was a belief that these biosecurity measures lacked practicality and were difficult to implement due to financial implications and time constraints. Participants wanted to be more involved in the design of interventions and this should be embraced to give farmers agency and investment in industry targets. These issues will be explored in more depth below.

The UK poultry industry is highly integrated, with the top five integrator companies, who supply major supermarket retailers, accounting for ~80% of total UK production (29). Following the poultry industry's 2010 target to reduce *Campylobacter* in chicken meat at retail, the integration of the UK poultry meat supply chain has been effective in rolling out widespread biosecurity measures across broiler farms to achieve these goals (30). Participants commented upon the velocity of change and were frustrated by continual changes to these biosecurity requirements, such as control room barriers. The perceived rate of change in biosecurity practises within the industry and difficulty in controlling and predicting *Campylobacter* infection may have fuelled some of the scepticism and frustrations expressed by participants regarding *Campylobacter* and biosecurity measures. Participants were frustrated with not being able to reliably produce *Campylobacter-*free flocks compared with *Salmonella*-free ones. Industry *Salmonella* targets were easier to achieve as vaccination of broiler breeders is believed to have played a role in reducing *Salmonella-*positive broiler flocks (31, 32). Lapses in biosecurity are also more likely to result in the introduction of *Campylobacter* than *Salmonella* to broiler flocks (32). Allen and Lavau (30) conducted interviews across the UK poultry supply chain and encountered similar frustrations with predicting and controlling *Campylobacter* and the apparent randomness in whether a biosecurity intervention proves successful in preventing flock colonisation. Participants commented that the results of routine *Campylobacter* testing and a published league table could be both a positive and negative experience, depending on the nature of the results. However, these results also motivated them to open discourse with other farms, to increase collaboration and knowledge exchange, and to reflect on their own practises, leading to self-directed improvement. Stress, caused by thinning and other flock cycle events, was highlighted by participants as a risk factor for *Campylobacter* colonisation and major barrier to *Campylobacter* control.

Many studies have demonstrated that partial flock depopulation, or thinning, is a risk factor for a broiler chicken flock to become colonised with *Campylobacter* (33–36). It is not yet clear if the relationship between thinning and *Campylobacter* colonisation results from associated stressors, bird age, or the breach in biosecurity that occurs during catching. Despite the threat to biosecurity that catchers posed during thinning, there was little demand to stop this process amongst interviewees due to the associated negative financial effect. Previous studies have identified the widespread practise of thinning in the UK, and the lack of enthusiasm to stop thinning due to the economic impact (37, 38). Particularly on independent farms, other financial considerations, such as the cost of biosecurity measures, were described by participants to be a barrier to biosecurity compliance. Fraser et al. (37) found a clear inverse relationship between the willingness of farmers to adopt a biosecurity measure and its estimated cost. Furthermore, participants discussed the benefits of financial inducements or penalties for *Campylobacter* results. Fraser et al. (37) concluded that this, or possibly a policy decision with legal ramifications, may be necessary to facilitate adoption of and ensure farmer compliance with biosecurity measures.

Audits and official enforcement of biosecurity measures (either conducted internally by an integrator company or externally by supermarkets and assurance schemes) were not framed positively by interviewees. Participants felt that there was too much auditing within broiler farming and admitted to only complying with certain biosecurity measures during audits. As previous studies have found, auditing, enforcement and direct observation only increase compliance in the short-term or create a tick-box exercise where people only comply when being observed and audited. The presence of visible CCTV cameras in broiler house control rooms have only been shown to improve biosecurity compliance in the short-term, with behaviour reverting to type within 6 months after installation (24). This study concurred with others that methods other than auditing are required to improve biosecurity compliance. There is a need to improve understanding of biosecurity measures by demonstrating why and how to apply them (24, 25, 39–41). Additionally, there needs to be an educational focus directed at explaining how diseases are introduced to a farm and the significance of each measure in terms of risk reduction, placing special emphasis on measures that are not applied despite their importance and effectiveness. Furthermore, current research on *Campylobacter* must be better communicated with all tiers of the broiler industry, including farm workers. Participants did not believe that *Campylobacter* has a detrimental effect on the health or welfare of chickens and felt that if *Campylobacter* did have a negative effect on broilers, then it would have been eradicated. *Campylobacter* has long been considered a commensal organism of broiler chickens. However, recent research has indicated that *Campylobacter* may cause disease in birds, negatively impacting upon their health and welfare and increasing the risk of hock burn and pododermatitis (42, 43). Arguably, biosecurity compliance may increase if broiler farm workers understood that *Campylobacter-*colonisation of flocks may have negative impacts on health, welfare, and performance.

Training and education were advocated by this study's participants, who believed that for farmers to comply with biosecurity measures it was necessary for them to “buy-in” and believe that the interventions will have an impact. It was clear from this study that education and knowledge exchange was crucially important to improve biosecurity compliance. These findings concur with a recent survey of the United States' broiler industry's understanding of *Campylobacter* interventions (40), which concluded that education and training programs were needed to improve the understanding of *Campylobacter* in broiler production, including the importance of on-farm biosecurity. However, training alone cannot be expected to solve the industry's issues with compliance. Millman et al. (27) investigated poultry catchers' understanding and experience of key biosecurity threats posed by poor compliance and the barriers to good biosecurity practise during thinning. The authors concluded that emphasising the importance of training was unlikely to result in gold standard biosecurity practise and reduction or removal of the barriers to implementing the required measure, such as through provision of extra time or equipment, may be a better aid to success. Catchers were described as in a “*Catch-22,”* where the time pressures of the job prevented them from complying with biosecurity protocols. What outsiders may have perceived to be the result of ignorance was seen by the catchers to be a necessary and conscious decision to adjust biosecurity protocols to complete the current job. In this study, time pressures were also a factor which affected reported biosecurity compliance. Participants admitted that during an emergency and the night-time, they were more likely to ignore biosecurity protocols, particularly with regards to wearing the correct PPE. Racicot et al. (25) found issues with biosecurity compliance regarding wearing PPE and handwashing, finding that these measures were often neglected, particularly for short visits (<17 min) and for those occurring during the afternoon. Previous studies have shown that farm design has been shown to play a role in compliance with biosecurity measures. For example, adequately positioned equipment (for example provisions for hand washing or PPE) is thought to contribute to enhancing and maintaining compliance (24). In this study, interviewees commented upon the importance of the practicality of biosecurity measures and the ease of their implementation to ensure compliance.

Conversely, Racicot et al. (24, 25) noted that some individuals simply seemed to willingly disregard the rules. This indicates that psychological characteristics may also be part of the problem and the authors advocated future investigation of personality traits, attitudes, and motivations (24). The effect of personality on a person's willingness to comply with biosecurity measures was discussed by interviewees. For example, participants felt that there were differences between different catching teams and that the personal interactions between the farm staff and the catching team influenced biosecurity compliance. Interestingly, Siekkinen et al. (44) found that female producers invest more financially in biosecurity than their male counterparts. Unfortunately, we were not able to investigate the role of gender on biosecurity compliance as only two women were interviewed in this study, which reflects the gender balance of the UK broiler farm workforce. There were differing views from participants as to the differences in biosecurity compliance between company-owned and contracted farms, with participants stating that compliance by company farms was better than on independently owned farms due to higher levels of oversight and enforcement. Hinchliffe et al. (45) describe a perception with the UK poultry industry that biosecurity is more effectively implemented within integrated production processes due to an ability to easily exercise control over the entire farm-to-fork chain. Similarly, East et al. (46) surveyed the level of adoption of a range of biosecurity procedures on Australian poultry farms and found a lower rate of adoption in independently owned farms, which was concluded to be due to the absence of guidelines imposed by a head office.

Ultimately, this study agreed with others that reasons for lack of compliance could not be boiled down to a lack of information or communication with personnel on biosecurity (24, 25, 27). Whilst lack of knowledge and training is an aspect of the problem, personal and farm characteristics are also determinants of compliance. Moreover, participants expressed a lack of autonomy and believed that their views and experience were often ignored in the design and implementation of interventions, which must be addressed. Interviewees expressed frustration with the lack of involvement they had in all decision-making processes, both on company and contract farms, and commented that before this study they had never been asked for their views on biosecurity interventions. Farmers possess tacit knowledge and experience that could be harnessed in the co-design and improvement of future interventions that may have positive and far-reaching effects on all aspects of the broiler industry. Allowing farmer contributions in this process will provide the “buy-in” required and give them agency to increase compliance and help the industry maintain its *Campylobacter* reduction targets. Biosecurity compliance may be improved by seeking to establish effective methods of communication, educating broiler farm workers about the importance of practises rated as too time-consuming, and by allowing more farmer input into the co-design of interventions.

This study suffered from some potential for bias during participant selection. Purposive sampling was used; two farms were selected through word-of-mouth and the other 14 farms were nominated by a major UK poultry integrator. The poultry integrator was asked to select farms with a range of internal biosecurity audit scores and *Campylobacter* results to ensure that a range of experiences and views were represented. Whilst 14 of the 16 participating farms were owned or contracted to one major UK poultry integrator, the main UK integrators require very similar on-farm standards and adhere to Red Tractor standards. Furthermore, ten participating farms were also independent, contract growers rather than company-owned farms, who may choose to contract grow for other poultry integrators. Thus, the views expressed are expected to be representative of the UK broiler industry. The results are also applicable to other intensively reared poultry species in the UK and similar rearing systems worldwide. Future work would benefit from exploring the views of broiler farm owners, managers and workers supplying other parts of the broiler chicken market and specific consumer demographics, such as the wholesale and Halal markets. Farms supplying the wholesale and Halal markets are less likely to be part of an integrated system that has undergone the recent overhaul to biosecurity measures, including reducing the number of thinning events to no more than one per flock, and Halal chicken meat has been demonstrated to have a higher *Campylobacter* prevalence than non-halal (47).

## Conclusion

In this study, we have shown there is a high level of recognition amongst broiler farmers of the importance of *Campylobacter* and other disease threats. All participants understood their responsibility in the reduction of *Campylobacter* colonisation of commercial broiler flocks. Participants' self-reported awareness and implementation of biosecurity measures has greatly improved following the industry-wide focus on *Campylobacter* control in broilers. There are frustrations with the industry's approach to tackling *Campylobacter* and the heavy burden of responsibility that has been put on interventions at the farm-level, particularly for a disease that is difficult to control and is not widely seen to detrimentally affect the health and welfare of broiler chickens. Compliance may be improved by establishing effective channels of communication with farmers to share current scientific research on *Campylobacter*. Additionally, more can be done to educate farmers with regards to the evidence-base supporting current biosecurity interventions. It is imperative that all players within the industry are asked to contribute to any decision-making process and are involved in the co-design of biosecurity interventions. Farmers are responsible for the implementation of biosecurity interventions and opportunities to develop and improve biosecurity measures and overall compliance may be achieved by utilising co-design approaches with farmer input. It is crucial to harness farmers' valuable on-farm experience and to give them agency and investment in the industry's *Campylobacter* reduction targets. However, the emphasis within the interviews that the target to reduce *Campylobacter* has had a noticeable positive knock-on effect on the implementation of biosecurity within the broiler industry is very positive. The universal recognition of the benefit of this with regards to broiler health and welfare and other important targets, such as reducing antimicrobial usage, leaves a legacy of which the UK broiler industry can be proud.

## Data Availability Statement

The raw data supporting the conclusions of this article will be made available by the authors, without undue reservation.

## Ethics Statement

The studies involving human participants were reviewed and approved by University of Liverpool Veterinary Research Ethics Committee, University of Liverpool (Reference VREC478). The participants provided their written informed consent to participate in this study.

## Author Contributions

NW conceived and designed the study and advised on all aspects of the analysis. AR contributed to study design, performed the data collection and analysis, and wrote the paper. RC advised on study design and data collection, analysis, and interpretation. All authors contributed to manuscript revision and approved the submitted version.

## Funding

This work was supported by a Biotechnology and Biological Sciences Research Council Doctoral Training Partnership Studentship (grant no. BB/J014516/1 to AR). The research materials supporting this publication can be accessed by contacting the corresponding authors.

## Conflict of Interest

The authors declare that the research was conducted in the absence of any commercial or financial relationships that could be construed as a potential conflict of interest.

## Publisher's Note

All claims expressed in this article are solely those of the authors and do not necessarily represent those of their affiliated organizations, or those of the publisher, the editors and the reviewers. Any product that may be evaluated in this article, or claim that may be made by its manufacturer, is not guaranteed or endorsed by the publisher.
