# Supplementary material for: The Role of Biosecurity in the Control of Campylobacter: A Qualitative Study of the Attitudes and Perceptions of UK Broiler Farm Workers
Source: Front Vet Sci. 2021 Dec 21;8:751699. doi: 10.3389/fvets.2021.751699 (PMC8724210; doi:10.3389/fvets.2021.751699)
Supplement: Supplementary file 1 [file Table_1.DOCX]

**The Role of Biosecurity in the Control of *Campylobacter*: A Qualitative Study of the Attitudes and Perceptions of UK Broiler Farm Workers**

Alexandra Royden, Robert Christley, Alison Prendiville & Nicola J. Williams

**Supplementary Material: Interview Codebook (Exported from NVivo)**

| Name | Description | Files | References |
| --- | --- | --- | --- |
| **Biosecurity Compliance** | Implementation and compliance with biosecurity measures | 2 | 25 |
| Other Contributing Factors | Factors affecting biosecurity compliance other than protocols resulting in requirement and enforcement | 0 | 0 |
| Comparisons with Others | Comparisons made by participants between themselves and others (individuals and sites) | 4 | 13 |
| Farmer Embarrassment |  | 2 | 6 |
| We have it easier |  | 1 | 1 |
| We have it harder |  | 4 | 7 |
| We're better |  | 7 | 14 |
| We're worse |  | 3 | 6 |
| Knowledge | Farmer knowledge regarding biosecurity practices | 3 | 20 |
| Agricultural Training | Comments on formal agricultural training | 1 | 3 |
| Biosecurity Training | Comments on training in biosecurity | 9 | 25 |
| Change Mindset | The effect of training on changing mindsets and therefore practices relating to biosecurity | 1 | 4 |
| Knowledge about biosecurity |  | 4 | 10 |
| Knowledge about Campylobacter |  | 6 | 32 |
| Knowledge Exchange | Effect of knowledge exchange between different parties on implementation of biosecurity practices | 1 | 5 |
| Lack of Knowledge | How the participant’s lack of knowledge or a universal lack of knowledge is affecting them/the farm/biosecurity. | 5 | 18 |
| Pre-existing knowledge | Knowledge or education gained from life/training/schooling prior to working for relevant farm/company. | 5 | 13 |
| Risk reduction vs eradication | Is biosecurity about reducing the risk of Campylobacter colonisation or eradicating it entirely? | 5 | 10 |
| Motivation | Motivation to implement biosecurity measures | 4 | 36 |
| Blame & Guilt | Looking for reasons that a failure in biosecurity or Campylobacter-positive flock is not their fault. Evidence of guilt that they feel responsible for flock positivity. | 1 | 21 |
| It's out of my control | Belief that cannot control Campylobacter colonisation of broiler flocks | 8 | 48 |
| Desire to Leave | Participants expressing a desire to leave their current role | 4 | 13 |
| Desire for another career path | Participants expressing a desire to leave their current role for another career | 2 | 5 |
| Retirement | Participants expressing a desire to leave their current role for retirement | 1 | 3 |
| Differences between farms and individuals | Differences between farms and individuals that are reflected in or result in differences in biosecurity compliance | 12 | 35 |
| External factors | Other things that motivate people to work, e.g. kids, debt, family, etc. These may increase or decrease job motivation. | 1 | 3 |
| Children |  | 1 | 2 |
| Spousal Responsibilities |  | 1 | 1 |
| Work-Life Balance | Desire for leisure time | 1 | 2 |
| Financially Motivated | Effect of money on participants’ performance in their role or on their compliance with biosecurity practices. | 5 | 10 |
| Frustrations | Frustrations which impact motivation in job/implementing biosecurity practices | 8 | 32 |
| Don't understand farms | External parties lack understanding which impacts motivation | 5 | 7 |
| Farm Issues | On-site issues which lead to frustrations and detrimentally affect motivation | 2 | 5 |
| Not being listened to | Feeling that one’s concerns are not being listened to or acknowledged appropriately, which detrimentally affects motivation | 1 | 4 |
| Increased Motivation | Factors which improve motivation for role/biosecurity | 3 | 10 |
| Competition with other farms | Informal and formal competition between farms. May include league tables and scoring systems used by integrators. | 2 | 9 |
| Desire to improve |  | 7 | 10 |
| Career Progression |  | 2 | 9 |
| Farm Improvement |  | 3 | 8 |
| Self-improvement |  | 1 | 8 |
| Doing it for the birds | Motivated to comply with biosecurity to benefit the broiler flocks under their care | 1 | 1 |
| Good Contract or Company | Expressed opinion that they like working for a company or like the company they are contracted to. | 6 | 12 |
| Job satisfaction |  | 2 | 4 |
| Pride in Work |  | 1 | 2 |
| Support | Support from other staff or management | 2 | 8 |
| Useful or Positive Feedback | Feedback that is considered useful and helpful to day-to-day running of farm or for personal/farm improvement | 6 | 15 |
| Lack of Motivation | Factors affecting motivation to implement biosecurity practices | 3 | 17 |
| Age |  | 1 | 2 |
| Boredom and Repetitiveness |  | 2 | 2 |
| Job Difficulties |  | 4 | 11 |
| Not Enough Staff |  | 2 | 9 |
| Lack of Control |  | 3 | 20 |
| Lack of Feedback and Communication |  | 5 | 17 |
| Lack of Time-Off |  | 4 | 6 |
| Negative Feedback |  | 3 | 9 |
| Personal Relationships | How relationships between teams etc affect motivation to perform tasks correctly. | 3 | 15 |
| Stress | Effect of stress on motivation to perform job and/or implement biosecurity practices | 3 | 12 |
| Struggling to Control Campylobacter | Stress attributed to attempts to control Campylobacter | 2 | 9 |
| Attempts to successfully control Campylobacter | Where attempts to control Campylobacter have been successful under stressful circumstances | 1 | 10 |
| Problems with Implementation | Problems with implementing biosecurity measures | 5 | 50 |
| Bird Stress | Effect of bird stress on Campylobacter colonisation of broiler flocks | 8 | 25 |
| Economic Pressure | Effects of biosecurity practices on profit margins | 9 | 19 |
| Failure to Implement | Failure to implement biosecurity measures | 8 | 34 |
| Lack of Support | How does a lack of support affect implementation of and compliance with biosecurity practices | 5 | 12 |
| Asking and Not Getting | Asking for assistance with implementation and not receiving it | 2 | 4 |
| Time Pressure | Lack of time results in pressures on biosecurity practices | 12 | 43 |
| Time Wasters | When biosecurity measures are time-wasting | 4 | 6 |
| Time-Savers | Actions taken to save time when implementing biosecurity measures | 2 | 2 |
| Successful Implementation | Successful implementation of biosecurity measures | 5 | 35 |
| Biosecurity as a habit | When biosecurity becomes second nature and habitual | 7 | 13 |
| Ease of Implementation | Measures or circumstances that make biosecurity practices easy to implement | 3 | 7 |
| Requirement and Enforcement | Enforcement of biosecurity measures | 3 | 14 |
| Biosecurity Audit | Use of biosecurity audits to improve compliance with biosecurity practices | 9 | 15 |
| Enforcement, Audits and Checks |  | 5 | 11 |
| In-House Checks | Internal or on-site enforcement | 4 | 15 |
| Campylobacter Audit | Use of Campylobacter audits to improve compliance with biosecurity practices | 12 | 30 |
| It's the Law | Legal requirement to carry out certain on farm practices | 2 | 2 |
| Pressure to correctly implement | Effect of pressure to correctly implement biosecurity measures | 6 | 22 |
| Obligation | Feeling of obligation to correctly implement biosecurity measures | 3 | 3 |
| Tick Box Exercise | Implementing biosecurity measures feels like a ‘tick-box exercise’ | 2 | 5 |
| Too much enforcement | Belief that there is too much enforcement which has a detrimental impact on biosecurity compliance, farm/flock performance, staff morale, etc | 5 | 10 |
| Red Tractor | Effect of Red Tractor on biosecurity compliance | 4 | 5 |
| Salmonella Testing | Salmonella testing of broiler flocks | 4 | 8 |
| **Biosecurity Issues and Improvements** |  | 0 | 0 |
| Biosecurity Practices & Risk Factors |  | 2 | 4 |
| Barriers | References to control room barriers | 11 | 31 |
| Chick Placement |  | 1 | 3 |
| Dead birds |  | 4 | 6 |
| Dirty Environment | Farm is a dirty environment with hygiene and biosecurity risks | 2 | 6 |
| External Visitors |  | 10 | 20 |
| Farm Traffic |  | 2 | 7 |
| Public Access |  | 2 | 3 |
| Flooding |  | 2 | 5 |
| Foot Dips |  | 4 | 11 |
| General Cleanliness |  | 3 | 5 |
| Hand Hygiene |  | 11 | 40 |
| Hatchery & Breeders |  | 5 | 13 |
| Litter & Bedding |  | 3 | 6 |
| Rotavating Litter |  | 2 | 4 |
| Maintenance |  | 4 | 9 |
| Neighbouring Farms |  | 3 | 4 |
| Other Farm Animals |  | 7 | 8 |
| Pests |  | 5 | 10 |
| PPE |  | 11 | 40 |
| Relief Staff |  | 3 | 8 |
| Shed-Specific Equipment |  | 5 | 11 |
| Spread of Disease |  | 1 | 1 |
| Proximity of Farms |  | 4 | 6 |
| Staff Members |  | 4 | 4 |
| Stocking Density |  | 1 | 1 |
| Terminal Disinfection |  | 7 | 19 |
| Thinning & Catching |  | 14 | 41 |
| Vehicle Disinfection |  | 10 | 16 |
| Ventilation |  | 2 | 8 |
| Water |  | 2 | 4 |
| Weather & Seasonality |  | 7 | 20 |
| Wildlife |  | 6 | 11 |
| Yard Surface | Presence of concrete apron and effect on Campylobacter colonisation | 3 | 4 |
| Potential Improvements | Potential improvements to biosecurity measures on broiler farms | 10 | 45 |
| **Campylobacter in Vogue** | Reference to how quickly the biosecurity changes have happened | 11 | 23 |
| Everybody's Talking about Campy | Campylobacter is the “in thing” and the current problem to be solved | 5 | 7 |
| Something else will come along | References to Campy being the topic of the moment or ‘in vogue’, but something else will replace it. It was Salmonella, now Campylobacter, etc. | 4 | 5 |
| **Importance of Biosecurity** | Importance of biosecurity in preventing Campylobacter colonisation of broiler flocks | 14 | 59 |
| General Discussion |  | 0 | 0 |
| Animal Health & Welfare Pressure | Pressure to improve health and welfare. Impacts on biosecurity practices and/or prevention of Campylobacter colonisation on health and welfare. | 3 | 7 |
| Feed |  | 3 | 4 |
| Feed Withdrawal | Feed withdrawal particularly around thinning and depletion | 3 | 4 |
| Health | Health issues | 4 | 8 |
| Hock Burn and Pododermatitis |  | 1 | 7 |
| Medication | Antibiotics and/or prescribing practices | 1 | 1 |
| Performance | Performance of broiler flocks | 4 | 12 |
| Welfare | Welfare of broiler flocks | 7 | 19 |
| Antibiotic Usage | Importance of biosecurity on antibiotic usage | 3 | 4 |
| Importance of other diseases | Importance of biosecurity on other diseases | 14 | 28 |
| Knock-on effect | Effect of improving biosecurity for the control of Campylobacter on other outcomes | 2 | 4 |
| The Legacy of Campylobacter Control | Long term impacts on the UK broiler industry from targets to control, reduce and prevent Campylobacter colonisation of broiler flocks | 3 | 6 |
| **Power and Responsibility** | Who is responsible for preventing and reducing campylobacteriosis? | 5 | 26 |
| Brexit | Topics relating to Brexit | 8 | 13 |
| Consumer Responsibility |  | 8 | 17 |
| Consumer Awareness |  | 10 | 30 |
| Everybody has a role | Everyone in the industry has a role to play in reducing campylobacteriosis | 1 | 1 |
| Farmer Responsibility | What are farmers' responsibilities with regards to Campylobacter and biosecurity | 7 | 14 |
| Only so much a farm can do | There is a limit to the amount that can be done on farm to reduce Campylobacter colonisation of broiler flocks | 3 | 5 |
| Industry Responsibility | Industry responsibility to prevent campylobacteriosis | 7 | 16 |
| Food Security | Responsibility of industry to ensure food security | 1 | 1 |
| Industry Motivation | Why the industry are motivated to solve Campylobacter (and other issues) | 1 | 3 |
| Financial Considerations | Industry’s financial considerations with controlling Campylobacter | 1 | 2 |
| Industry Embarassment | Industry’s embarrassment of Campylobacter levels in chicken meat | 1 | 3 |
| Legislative Demands | Legislative demands to motivate industry to reduce Campylobacter prevalence in chicken meat | 1 | 1 |
| Supermarket Demands | Supermarket demands to motivate industry to reduce Campylobacter prevalence in chicken meat | 5 | 12 |
| Media Responsibility | Media responsibility for public perception of poultry industry and campylobacteriosis | 5 | 9 |
| Press Coverage | Media involvement and portrayal of the industry | 2 | 5 |
| Researchers Responsibility | Scientific community’s responsibility for reducing campylobacteriosis | 2 | 2 |
| Slaughterhouse Responsibility | Slaughterhouse responsibility for reducing campylobacteriosis | 8 | 12 |
| Supermarket Responsibility | Supermarket responsibility to prevent campylobacteriosis | 3 | 10 |
| **Scepticism and Controversy** | Lack of belief in necessity for biosecurity implementation, or frustration with lack of knowledge and information available | 8 | 40 |
| About Campylobacter | Scepticism and controversy about the bacterium itself | 0 | 0 |
| Campylobacter doesn't affect broilers | Discussions surrounding belief that Campylobacter does not have a detrimental effect on broilers | 2 | 3 |
| Frustrating Bug | Frustrations with controlling Campylobacter | 8 | 23 |
| Multifactorial Problem | Belief that there is not one solution to Campylobacter control but a suite of measures required to prevent colonisation of broiler flocks | 3 | 7 |
| Things can be too clean | Discussions around concept that reducing bacterial contamination too much can have a negative impact on individuals and society as a whole | 1 | 1 |
| Unseen Threat | The invisible presence of Campylobacter in the broiler farm environment | 2 | 2 |
| Won't Eradicate Campylobacter | Belief that it isn’t possible to eradicate Campylobacter from broiler flocks | 6 | 11 |
| Nothing More We Can Do | Belief that these is nothing more that can be done on broiler farms or in the broiler industry to prevent Campylobacter colonisation of broiler flocks | 4 | 10 |
